# More evidence is needed to improve molecular HIV surveillance for cluster detection and response

**DOI:** 10.1038/s43856-025-01202-0

**Published:** 2025-11-14

**Authors:** Anne LR Schuster, Juli Bollinger, Gail Geller, Susan J. Little, Sanjay R. Mehta, Travis Sanchez, Jeremy Sugarman, John FP Bridges

**Affiliations:** 1 Department of Biomedical Informatics, The Ohio State University College of Medicine, Columbus, OH USA; 2 Berman Institute of Bioethics, Johns Hopkins University, Baltimore, MD USA; 3 School of Medicine, Johns Hopkins University, Baltimore, MD USA; 4 Division of Infectious Disease, University of California San Diego, San Diego, CA USA; 5 Department of Epidemiology, Rollins School of Public Health, Emory University, Atlanta, GA USA; 6 Department of Health Behavior and Society, Bloomberg School of Public Health, Johns Hopkins University, Baltimore, MD USA

**Keywords:** HIV infections, Epidemiology

## Abstract

**Background:**

Molecular HIV surveillance (MHS) is used in the U.S. to inform public health prevention and intervention activities aimed at helping end the HIV epidemic. Its application in this context is currently challenged by ethical, legal, and social concerns, with gaps in understanding how end users weigh these issues. We sought to identify the preferences of MHS end users for improving MHS.

****Methods**:**

End users completed 12 choice experiment tasks evaluating five attributes of MHS for cluster detection and response. We generated a choice model using conditional logit and report results as relative attribute importance scores, comparing them to attitudinal data from close-ended questions. Responses to open-ended questions provide additional context on areas for MHS improvement.

**Results:**

We report findings from 55 of 90 potential end-users who felt capable and agreed to participate. End users include researchers (n = 28) and public health practitioners (n = 27); their preferences do not differ significantly (p = 0.174) so their responses are combined. The highest weight is placed on certainty of MHS benefit (38%), followed by depth of HIV sequence sampling (26%). Lower weight is given to reducing stigma (20%) and personalized inferences (10%) and communication (6%). End users highlight improving MHS through implementation support, community engagement, transparent communication, intervention assistance, risk minimization, and impact assessment; researchers stress the need for better data access.

**Conclusions:**

End users place the highest value on having more evidence of MHS benefits. Improving MHS requires attention to implementation support, community engagement, transparent communication, intervention assistance, risk mitigation, impact assessment, and data access.

## Plain language summary

Molecular HIV surveillance (MHS) may aid in efforts to prevent onward HIV transmission, but ethical, legal, and social issues are associated with its use in this context. This study explored how researchers and public health professionals, termed as “end users”, would like to see MHS improved. The team conducted a choice experiment with end users. The most important factor for participants was having clear evidence that MHS provides real benefits. They also valued better support for using MHS in public health efforts, reducing risks, transparent communication, community involvement, and measuring impact. The findings highlighted the need for better access to data, which in turn may help guide more ethical and effective use of MHS in the future.

## Abstract (web summary)

Schuster et al. engaged researchers and public health professionals working in molecular HIV surveillance (MHS) to assess how they value strategies to improve its use. Participants indicate that the greatest emphasis should be placed on providing certainty of benefit, considering the range of ethical, legal, and social implications of MHS.

## Introduction

Molecular HIV surveillance (MHS) is being used in the US to monitor and intervene in the spread of HIV^1–5^. MHS utilizes HIV gene sequences obtained from routine clinical drug resistance testing among people living with HIV. It examines genetic relationships between HIV sequences and has provided insights into risk factors and patterns of drug resistance^6–9^, the dynamics of HIV spread during outbreaks^10–13^, and the evolution of transmission networks over time^14–16^. More recently, MHS has been used to detect and respond to emerging HIV clusters with the intent to deliver more focused and tailored interventions to prevent new infections^5,17,18^. In 2018, the US Centers for Disease Control and Prevention (CDC) mandated that all CDC-funded health departments use MHS in their efforts to detect and respond to clusters^19,20^ as part of an initiative called Ending the HIV Epidemic^21^.

The use of MHS for cluster detection and response has raised important social, ethical, and legal concerns. Numerous advocacy organizations have opposed it, with some calling on the CDC to halt its use^22^. They argue that the personal and medical information of people living with HIV are being leveraged for MHS without adequately involving them in program planning, a lack of transparency regarding the use of their individual data, and a lack of informed consent for using these data^23,24^. There are also concerns about the lack of direct and quantifiable benefits of MHS^5,25^, the risk of exacerbating stigma against people living with HIV and historically marginalized populations, and the potential to infer the directionality of HIV transmission between a pair of persons living with HIV, which might increase the risk of criminal or civil litigation^25–30^.

In light of these concerns, the Presidential Advisory Council on HIV/AIDS (PACHA) passed a non-binding resolution in 2022 calling for public health agencies to take substantial actions^31^. This includes more effectively engaging communities about MHS, gathering evidence on its impact, providing plain language notifications about it, ensuring informed consent, and reviewing and repealing laws that disproportionately affect people living with HIV, particularly those related to HIV criminalization. While some studies have explored these concerns and recommendations among different stakeholder groups have been proposed^32–36^, there is a need for a comprehensive and systematic assessment of how to respond effectively to these concerns. As such, our multidisciplinary team conducted a multi-phase research project entitled, “Study of Ethics and Stakeholder Attitudes toward Molecular Epidemiology” (SESAME) to begin to overcome this deficit and thereby inform the analysis of the related ethical, legal, social and policy issues associated with the use of MHS for cluster detection and response. Previous SESAME studies have explored various aspects of HIV molecular epidemiology, including perspectives of it from people living with HIV or who are at risk of acquiring HIV^32^, user experiences with it in research and public health^37^, end users’ prioritization of its ethical concerns such as increased risk of legal prosecution^38^, perceptions of MHS among men who have sex with men^39^, the impact of disclosing the use of antiretroviral resistance testing results for MHS^40^, clinician perspectives on disclosing public health uses of HIV antiretroviral resistance testing results^41^, and the risks of HIV transmission attribution from simulated sequencing data^42^. The current study builds on this foundation.

In this study, we sought to identify the preferences of MHS end users for improving MHS practices for cluster detection and response. In addition, we compared the preferences of MHS end users with their attitudes towards improving MHS practices for cluster detection and response to explore the concordance of the findings. We find that end users place the highest value on having more evidence of MHS benefits. Improving MHS requires attention to implementation support, community engagement, transparent communication, intervention assistance, risk mitigation, impact assessment, and data access.

## Methods

We engaged end users through a survey including close- and open-ended questions and a choice experiment to elicit end-user preferences. A choice experiment is a theory driven method grounded in psychology^43^ and economics^44^. Choice experiments have been used to analyze the preferences of stakeholders and policymakers to inform HIV programs, policies, and interventions^45^. This method was chosen to provide actionable insights into end-user preferences, aligning with our aim to identify areas for improving MHS for cluster detection and response. The close-ended questions were included to facilitate end-user understanding and to enable comparison of results from the choice experiment. Results were contextualized with open-ended questions about additional areas of improvement for MHS.

### Population

End users invited to participate in our study included researchers with expertise in MHS and practitioners from U.S. public health departments who had experience with oversight or implementation of MHS for surveillance and/or public health responses to HIV. Participants were identified through their authorship of peer-reviewed publications, presentations at major HIV conferences^46,47^, leadership roles in national societies/centers, publicly available lists of HIV-focused public health officials^47,48^, and/or the project’s Expert Advisory Board members. All potentially eligible end users were invited to participate in the survey via email. End users who opted in to participate were sent an individual online survey link administered through Qualtrics (Qualtrics, Provo, UT). The survey was fielded from May to June 2023.

### Survey

**Fig. 1:**
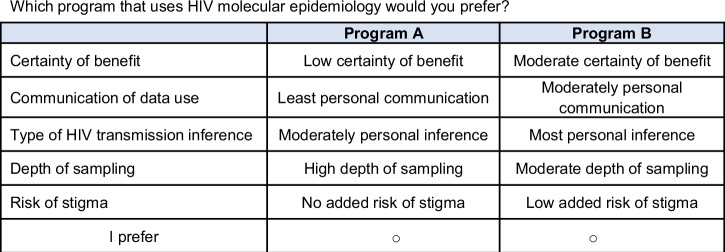
Example choice task. This figure presents an example of the choice tasks asked as part of the choice experiment that included five attributes with three levels each of MHS programs employing HIV molecular epidemiology.

The choice experiment was developed following good practices^49^. This involved synthesizing evidence from the literature (see Supplementary Methods for detailed information on U.S. MHS data sources and analytic approaches), engaging experts to identify possible attributes for inclusion, and then pre-testing and pilot-testing the instrument. Five attributes and levels of MHS programs employing HIV molecular epidemiology were selected (see Supplementary Table 1 for attribute descriptions): certainty of benefit (low, moderate, high benefit^50^); communication about data use (least personal defined as information on website, moderately personal defined as direct notification, most personal defined as discussion as part of informed consent process); types of HIV transmission inference (least personal defined as network transmission, moderately personal defined as individual transmission linkage, most personal defined as individual transmission directionality); risk of stigma (no added risk, low added risk, moderate added risk); and depth of sampling of HIV sequences (low defined as 40% sampling depth, moderate defined as 60% sampling depth, high defined as 80% sampling depth). Depth of sampling was described as the proportion of people living with HIV in a given region with viral sequences available for analysis and it was noted that depth of sampling might influence the detectability of networks experiencing rapid HIV transmission. The choice experiment asked end users to choose among pairs of programs characterized by these five attributes. An example choice task is presented in Fig. 1. NGene (ChoiceMetrics, Melbourne, Australia) was used to create the experimental design, which consisted of twelve choice tasks.

One set of close-ended questions assessed end users’ attitudes about the importance of each attribute for the design of MHS programs (important, somewhat important, not important). Open-ended questions inquired about additional issues that end users considered important to address in designing MHS programs. The survey also included closed-ended questions on end users’ primary role (researcher or public health practitioner), number of years working in their field (<5 years, 5–10 years, >10 years), general awareness (not aware, somewhat aware, very aware) and concerns (not concerned, somewhat concerned and very concerned) of ethical, legal and social implications (ELSI) regarding MHS among people in their profession. Another set of close-ended questions asked end-users to express whether there was concern (yes/no) about the following 11 specific ELSI issues regarding MHS among people in their profession: limited evidence of benefits; lack of individual consent; lack of directly disclosing data use; lack of an opt-out option; limited resources for other activities; inferring source of HIV transmission; inferring directionality of HIV transmission; increased risk of harm towards individuals; and increased risk of stigma towards groups. The following standard debriefing questions were asked about the choice experiment: the ease of understanding it, the ease of answering it, its relevance to them, and its reflection of their preferences.

### Data analysis

Analysis of the choice experiment was guided by good research practices^51^. A conditional logit model was used to analyze the choice decisions from the choice experiment. Choice decisions were treated as a binary dependent variable and regressed on the level of change in the MHS attributes. The attributes were treated as continuous variables to generate more parsimonious estimators^52^. A Swait-Louviere test was used to test for the poolability of end users^53^. Relative attribute importance estimates were generated from the conditional logit results, where higher estimates indicate greater intensity of preferences. Percent endorsement was calculated for the choice experiment debrief questions, where endorsement was defined as an agree/strongly agree response.

Descriptive statistics were generated for end-users’ attitudes towards the five MHS attributes, where responses were: dichotomized (important/not important), summarized using relative frequency statistics, and compared to the estimates of relative attribute importance from the choice experiment using Pearson’s $\rho$. Descriptive statistics were generated for end-user characteristics and their concerns about specific ELSI issues where categorical variables were summarized using frequency statistics and compared using chi-square tests with two-sided hypothesis. All quantitative analyses were conducted using Stata SE version 17 (StataCorp LLC).

Data from the open-ended questions were analyzed using reflexive thematic analysis according to the following phases: (1) becoming familiar with the data; (2) generating codes; (3) generating themes; (4) reviewing themes; and (5) defining and naming themes^54–56^. The research team became familiar with the data by reviewing, cleaning, and removing any identifiable information. The survey team (ALRS and JFPB) met to review the initial codes, establish a refined codebook, discuss initial themes, and define and name final themes. Thematic findings were illustrated with representative quotes to support further understanding.

### Oversight and ethics

The development of the survey was informed by engagement with experts in the use of MHS in public health and research. These individuals were selected based on their leadership, experience publishing guidance on MHS methods, practical application of MHS, and/or knowledge of ethical considerations of MHS in both research and public health contexts. This expert engagement was reviewed and deemed non-human subjects research by the Johns Hopkins Medicine Institutional Review Board (reference number IRB00354016) under U.S. Department of Health and Human Services (DHHS) or Food and Drug Administration (FDA) regulations. The conduct of the survey was reviewed and also deemed non-human subject research by the Ohio State University College of Medicine Institutional Review Board (reference number 2022E1207) based on these regulations. Accordingly, there was no formal requirement to obtain written informed consent. However, only individuals who agreed to help with survey development did so, thus implying their consent to participate. In addition, the survey introduction provided essential information to potential participants about the survey, including the purpose of the survey, the estimated time to complete the survey, and the option to stop the survey at any time as well as assurances that participation was voluntary and individual responses would be treated confidentially. The survey introduction concluded with a statement: “If you are willing to participate, please click the ‘Next’ button at the bottom of this page.” Completion of the survey was an indication of implied consent. An advisory board also provided input on the project.

### Reporting summary

Further information on research design is available in the Nature Portfolio Reporting Summary linked to this article.

## Results

We report findings from 55 of 90 potential end users who opted in to the survey (response rate = 61%), including end users engaged in research (n = 27) and public health (n = 28). Table 1 summarizes characteristics of the end users. The two groups differed in their years of work experience; more public health practitioners (40%) than researchers (10%) reported working in their field for five years or less (p = 0.01). Both groups demonstrated similar levels of awareness and concern regarding ELSI issues related to MHS.

**Table 1:** Characteristics of MHS end users

Characteristic, mean (se)	Researcher (n = 27)	Public health (n = 28)	p-value
Years worked in their field			
0–5	0.080	0.370	0.01
	(0.05)	(0.09)	
6–10	0.160	0.111	0.61
	(0.07)	(0.06)	
More than 10	0.760	0.519	0.07
	(0.09)	(0.09)	
Perceived awareness of ELSI			
Not aware	0	0	-
	(0)	(0)	
Somewhat aware	0.296	0.214	0.49
	(0.09)	(0.08)	
Very aware	0.704	0.786	0.49
	(0.09)	(0.08)	
Perceived concern about ELSI			
Not concerned	0.111	0.107	0.96
	(0.06)	(0.06)	
Somewhat concerned	0.666	0.571	0.48
	(0.09)	(0.10)	
Very concerned	0.222	0.321	0.42
	(0.08)	(0.09)	
Number out of 11 ELSI concerns	6.481	4.857	0.05
	(0.58)	(0.54)	

*ELSI* ethical, legal, and social implications.

**Fig. 2:**
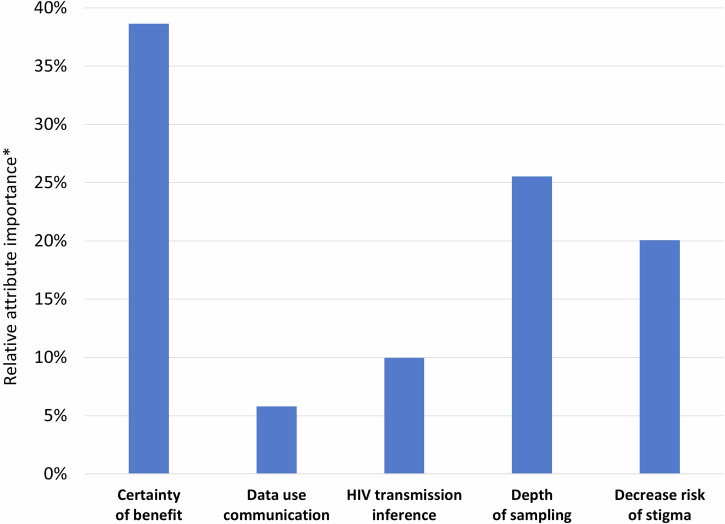
Ways to improve MHS based on choice experiment. This figure shows the relative attribute importance of five attributes of MHS programs. *Relative attribute importance sums to 100%.

The results of the Swait-Louviere test failed to reject the null hypothesis that the estimated preferences between end-user groups (researchers and public health practitioners) were the same (p = 0.174). This indicated that the data from the two groups could be pooled (Supplementary Fig. 1). Figure 2 shows the relative importance of MHS attributes. End users indicated it was most important to increase the certainty of MHS benefit (38%). This was followed by having greater depth of sampling (as defined above) of HIV sequences (26%) and then decreased risk of stigma (20%). They placed less importance on the type of HIV transmission inference (10%) and on personalized communication to individuals about the uses of their data for MHS (6%).

**Fig. 3:**
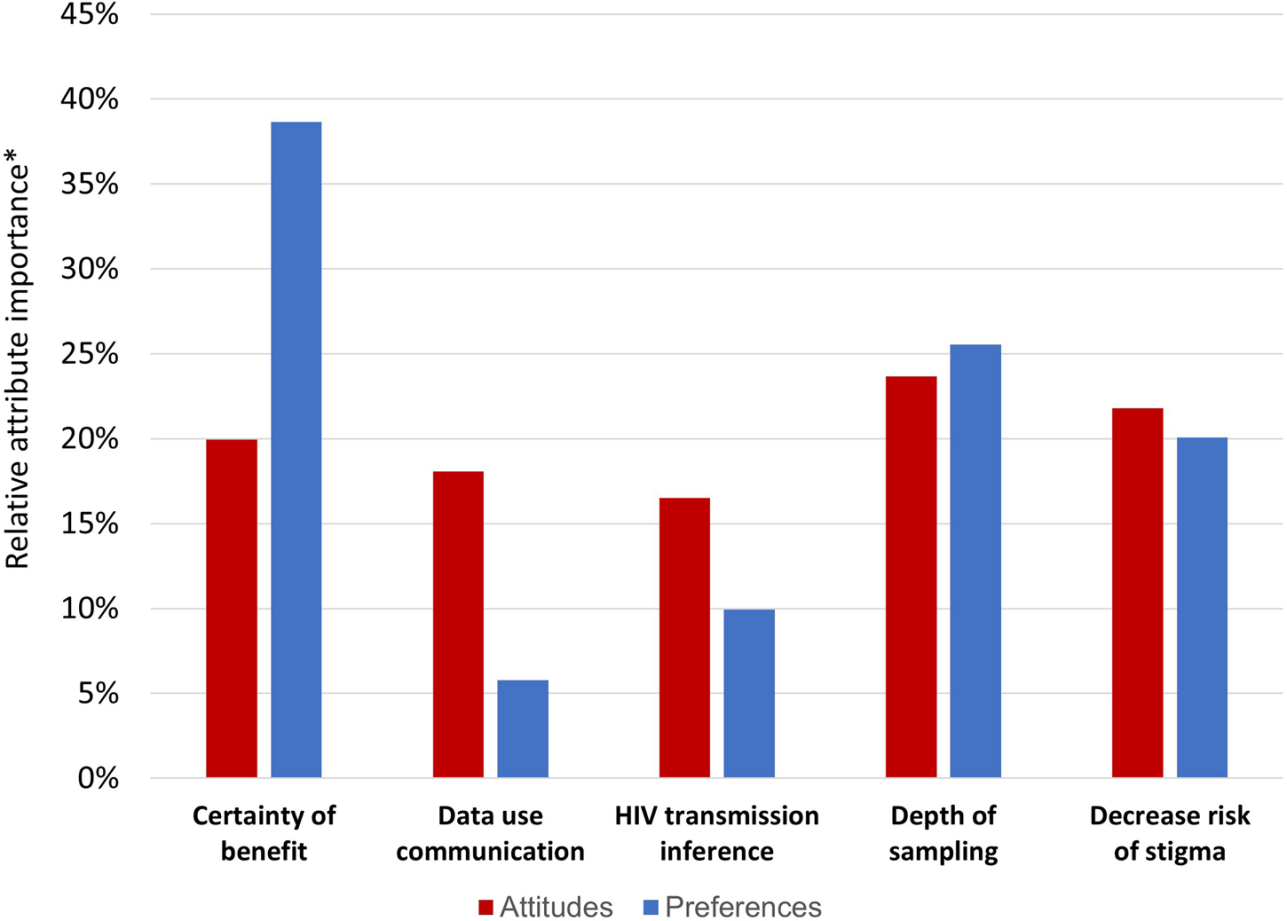
Comparison of attitudes and preferences to improve MHS. This figure shows relative attribute importance of attitudes and preferences about five attributes of MHS programs. Attitudes were assessed using close-ended questions about the importance of each attribute and preferences were assessed using a choice experiment. *Relative attribute importance sums to 100%.

There was concordance ($\rho$=0.55) between end users’ preferences and their attitudes toward MHS attributes (Fig. 3). End users’ relative attitudes towards the attributes ranged from 17% to 24%, which suggests relatively uniform views across all attributes. Most end users found the choice experiment was easy to understand (76%), reflective of their preferences (61%), and relevant (67%); though fewer found it easy to answer (40%).

### Additional insights

**Fig. 4:**
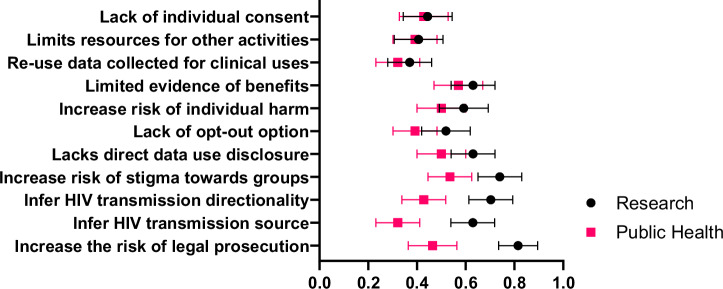
Comparison of ELSI concerns about MHS among end users. This figure shows proportion of end users with ELSI concerns. Concerns were stratified by end users. ELSI concerns were assessed using binary close-ended questions.

Figure 4 presents how end users perceived 11 specific ELSI concerns related to MHS based on close-ended questions. Some differences emerged between researchers and public health practitioners even though their pooled preferences about MHS attributes showed agreement. Researchers expressed significantly greater concern about MHS enabling inferences about the directionality of HIV transmission (70.3% vs. 42.8%, p = 0.04) and the source of transmission (62.9% vs. 32.1%, p = 0.02). They were also more concerned about the potential for MHS to increase the risk of legal prosecution (81.5% vs 46.4%, p < 0.01).

Over half of end users (63%) identified additional issues that they believed needed to be addressed to improve MHS practices. These issues fell into seven categories: data access; implementation support; intervention assistance; risk minimization, transparent communication; community engagement; and impact assessment. Table 2 summarizes these issues and includes representative quotes. Each category is elaborated on below.

**Table 2:** Additional issues to consider for improving MHS from open-ended responses

Themes	Description	Quotes
**Data access**	Technical aspects of data collection, management, and analysis, including handling missing data and integrating linked data.	“*The use of additional metadata (*STDs*) and demographics to better inform characteristics of the growing clusters” (*Researcher*); “More data is needed to develop real ground-breaking methods to advance our understanding of HIV transmission and evolution (*Researcher*)*
**Implementation support**	Practical aspects of executing interventions, including resource availability, data accessibility, and feasibility.	“*Public health data are not generally available in real time - if we are trying to identify clusters that are growing NOW, we need data collected within the last several months - not years.*” (Researcher)
**Intervention assistance**	Strategic guidance, planning and design of public health interventions informed by MHS data.	“*Define criteria to define priority clusters for intervention*” (Researcher); “*Capacity (not enough staff to do this activity) and resources to implement a fair, just, and effective program.*” (Public health practitioner)
**Risk minimization**	Strategies to mitigate potential risks from MHS results, including stigma, legal risks, and data protection.	“*Protection of data and results.*” (Researcher); “*The legal landscape of the jurisdiction in question, and data sharing policies that may place HIV+ individuals in legal jeopardy*” (Public health practitioner)
**Transparent communication**	Clear communication and transparency in the implementation of MHS programs, including from data collection and data use to data protection and results dissemination	“*Make sure that the public knows about the program’s benefits, and how data is collected, used and protected.*” (Public health practitioner); “*Having patients informed of the potential use of the test results by a trusted persons already involved in their care would lead to half the battle being won.*” (Public health practitioner)
**Community engagement**	Involving and earning the support of the community, including in use of MHS in program development and decision making.	“*Community awareness and engagement opportunities” (Researcher); “CABs representing the impacted communities need to be involved developing programs.*” (Public health practitioner)
**Impact assessment**	Demonstrate impact and efficiency of MHS programs like reducing HIV incidence and optimizing resources.	“*What is the contribution of MHS to reduced incidence?*” (Researcher); “*The trade-off risk of not performing molecular surveillance.*” (Public health practitioner)

Data access emerged as a key area for improvement, particularly among researchers. End users emphasized the need for broader access to data to advance understanding of HIV transmission and evolution, as well as to analyze the dynamics of local epidemics. Researchers called for resolving missing data issues, ensuring data quality, and collecting additional metadata—including information on other sexually transmitted infections, demographic characteristics, and social determinants of health—to strengthen analyses. They also highlighted the importance of improving the technical ease of conducting and interpreting analyses.

Implementation support was another issue that end users felt needed to be addressed. End users cited challenges such as limited resources, delayed availability of data and findings, and feasibility constraints, which vary across jurisdictions. Researchers highlighted that public health data are often not available in real-time, which poses a challenge for timely identification of growing clusters. Both groups stressed the need for faster reporting of analyses to inform interventions based on current information. Public health practitioners emphasized the need for adequate staffing and technical capacity to interpret and act on MHS findings. Some also noted that MHS activities can be time-intensive and difficult to implement without additional support, particularly in high-burden settings. Others pointed out that failing to conduct MHS may delay the identification of rapidly growing outbreaks, underscoring the importance of timely implementation.

Intervention assistance was seen as important for translating MHS findings into action. End users recognized that MHS may help optimize public health resources and ensure that individuals most in need receive timely interventions. However, they expressed a need for clearer guidance on how to apply MHS findings in practice. Respondents recommended developing criteria to define which clusters should be prioritized for intervention, noting that this is an area of ongoing discussion. Public health practitioners also highlighted the need for additional resources to support new cluster response activities. Some described efforts to use MHS to identify unmet social needs of individuals within clusters with the goal of connecting these individuals with appropriate services.

Risk minimization was another area of critical concern. End users identified potential risks associated with the use and reporting of MHS results, including legal implications. Researchers expressed the need for better quantification of sampling biases and improved understanding of analytical interpretations to reduce the risk of misinterpretation. Both researchers and public health practitioners called for data protection policies. Public health practitioners stressed the importance of considering the legal context of each jurisdiction, particularly where data sharing across state lines could place individuals at risk. Some researchers also emphasized that MHS should focus on the virus and public health interventions—such as testing, treatment, and care engagement—rather than on individual responsibility for transmission.

Transparent communication was viewed as necessary across all stages of MHS—from data collection to dissemination. End users recommended involving trusted care providers to explain MHS to patients and emphasized the need for public-facing communication to build awareness and trust. They urged using carefully crafted messaging, especially for more invasive programs, where attention should be paid to the intent of MHS, how data are derived and ethically used, and how the public benefits from the results.

Community engagement was seen as relevant to the success of MHS. Some end users expressed concern that current MHS efforts insufficiently incorporated community perspectives and emphasized that engagement must go beyond outreach to include meaningful participation of people living with HIV in decision-making processes. Several end users advocated for the involvement of Community Advisory Boards representing impacted communities to guide the development and implementation of MHS programs. Others stressed the importance of community endorsement and acceptance, noting that such support could improve the receptiveness of interventions among clients in transmission and associated risk networks. Additionally, end users highlighted the value of community-informed approaches that identify and address unmet social needs within clusters, suggesting that such strategies could enhance the effectiveness of public health responses.

Impact assessment of MHS programs was cited as an unmet need. End users emphasized the importance of evaluating whether MHS generates novel insights and contributes meaningfully to epidemic control beyond what can already be achieved through existing surveillance methods, such as analyses of demographic, care engagement, and viral suppression trends. While some public health practitioners cited their own experiences as evidence of the potential value of MHS, others called for more rigorous evaluation of its effectiveness and cost-benefit ratio. There was also concern that, despite the investments in MHS, there was limited interest in formal evaluations that could assess its impact, guide resource allocation, and inform program improvements.

## Discussion

This study contributes vital information to ongoing discussions about modifying MHS practices and policies from the perspectives of MHS end users, specifically researchers and public health practitioners^23,24,31,57^. By placing the most importance on determining if there is certainty of benefit, the findings demonstrate that MHS end users value answering a key question raised by advocates, scholars, and legal professionals, namely is “MHS worth the risk?”^58^ The risks being referred to include concerns about stigma, criminalization, and lack of disclosure about data use – issues that end users in our study were asked to consider directly or indirectly. It is worth noting that the findings are relative to one another, so while some attributes were weighted more highly, it does not imply that the other attributes are unimportant. We suggest instead that their relative value illustrates the critical need to evaluate the effectiveness of MHS and to weigh its demonstrated benefits and actual harms. The findings from our study can bridge conversations that inform the future of MHS and its use. The findings will be discussed in order of relative attribute importance to end users.

End users emphasized the importance of demonstrating the effectiveness of MHS for cluster detection and response. Notably, the certainty of benefit attribute was nearly 1.5 times as important as depth of sampling, the second most important attribute. Some end users expressed concern about a perceived lack of emphasis in evaluating the impact of MHS. They have articulated a desire for evidence demonstrating whether MHS contributes to reducing new HIV transmissions and improving HIV care outcomes, as well as for clarification of the mechanisms through which such effects may be achieved. These findings are consistent with knowledge gaps described in the literature^5,25^ and suggest that decision makers prioritize assessing the relative and attributable benefits of MHS on HIV transmission and/or HIV prevention and care outcomes. As they do so, it is also important to identify who should lead such efforts – whether it be researchers, public health practitioners, or joint partnerships between them.

Our findings indicate that end users valued greater depth of HIV sequence sampling, suggesting that enhanced sampling may yield more accurate and actionable insights. End users emphasized the importance of comprehensive data collection for identifying transmission patterns. This emphasis aligns with current policy that requires reporting of HIV sequence data–collected through routine clinical care—to state health agencies. The policy is based on the assumption that high-levels of sequence reporting are necessary to detect transmission clusters^20^. Arguably, high-quality and complete data are also likely to be essential for evaluating the effectiveness of MHS. End users may have also valued greater depth of HIV sequence sampling due to personal experiences or concerns about declining submission rates of HIV genetic sequences to public health authorities, as reported elsewhere^33,37^. Additionally, end users highlighted the need for robust data management systems to handle and analyze complex datasets, ensuring their integrity and utility.

Minimizing the risk of stigma was another priority for end users, which underscores their awareness of concerns raised by scholars and advocates regarding the potential for MHS to magnify the stigmatization of people living with HIV, individuals at risk of acquiring HIV, and their communities^26^. This is particularly salient given ongoing concerns that MHS studies may further position disproportionately affected communities as central to high-risk sexual networks in the U.S.^26,27,59^. Our findings indicate that end users recognize this potential risk as they emphasized the need for carefully crafted communication strategies, particularly when disseminating results or designing interventions based on MHS analyses. Some end users explicitly recommended the development of anti-stigma campaigns to accompany MHS efforts.

End users placed relatively less importance on the type of HIV transmission inference generated by MHS programs compared to other attributes. Yet, they echoed concerns raised by activists and legal experts regarding the risks of privacy breaches and criminalization^26,30,60^. This suggests the need for policies and practices that safeguard individual data and mitigate legal risks, particularly if MHS results could produce more personal transmission inferences. Researchers expressed greater concern than public health practitioners about the potential for MHS to increase the risk of legal prosecution, infer the source of HIV transmission, or infer directionality. However, public health practitioners were aware of the implications of generating such inferences and emphasized the importance of understanding the legal landscape of their jurisdiction, including data sharing policies that could jeopardize people living with HIV.

End users placed the least emphasis on personalized communication regarding the use of individuals’ data for MHS, relative to other MHS attributes. This is notable given the range of recommendations from stakeholders advocating for improved MHS communication practices, such as incorporating informed consent for the use of clinical data for MHS, allowing opt-outs from the uses of such data, and providing plain-language notifications to individuals living with HIV about the types of analyses conducted using their data^24,31^. Nevertheless, it is important to recognize that our findings reflected the perspectives of end users, specifically researchers and public health practitioners, and not necessarily those of people living with HIV, whose blood samples and information underpin MHS. It is plausible that the perspectives and priorities of end users may differ from those of people living with HIV, though this question was beyond the scope of the current study and warrants future investigation. Despite placing less importance on personalized communication, end users still expressed a need for clear and transparent messaging to alleviate concerns and foster broader acceptance of MHS. Further research may be needed to assess whether end users find existing publicly accessible resources adequate, which includes websites that explain data collection, usage, and protection. Additionally, end users noted the importance of engaging communities to inform the development of more ethical and acceptable MHS practices, thereby aligning with recommendations from prior studies^34,36,61^ aimed at building trust at the community level.

Our findings highlight the added value of using choice experiments to generate evidence that is useful for decision-making. The choice experiment identified a wider range of importance (6% to 38%) for MHS attributes compared to the relatively narrower range (17% to 24%) observed in the closed-ended attitudinal questions. This suggests that the choice experiment more effectively differentiated the relative importance of the attributes to expert end users. This distinction reflects several strengths commonly attributed to choice experiments^62^. One advantage is their ability to overcome limitations of traditional rating and ranking methods, such as variability in interpreting Likert-type scales, thereby enabling more precise and consistent elicitation of preferences^63–67^. Another strength lies in their capacity to capture more detailed information, allowing for clearer differentiation among preferences and producing higher-quality, and more precise results. Additionally, by requiring respondents to make trade-offs, choice experiments help identify nuances in preferences that might otherwise remain obscured^63,68–72^. Our findings align with previous studies^73,74^ and highlight the utility of choice experiments as a robust tool for eliciting preferences and contributing to a more accurate, comprehensive understanding of participants’ priorities.

We report several unique limitations and challenges with this study. First, there are few established methods for identifying and engaging professionals in U.S. public health departments. To address this, we implemented several approaches to do so systematically and comprehensively. Another challenge involved the varying timelines of cluster detection and response implementation across U.S. jurisdictions. While some public health departments adopted MHS early during the CDC’s pilot phase^5^, others are only beginning implementation. As a result, public health practitioners’ experiences with MHS are likely to differ by jurisdiction and our survey does not account for these variations. Additionally, not all end users responded to the open-ended question, and some responses were very brief. This may reflect that the choice experiment already captured their key concerns, or that time constraints limited their engagement. Nonetheless, including open-ended questions aligns with good practices for surveys incorporating choice experiments^49^. Finally, this study focused on the professional perspectives of end users of MHS, and it was beyond the scope of this study to include people living with HIV. Future research should systematically explore the perspectives of people living with HIV or those at heightened risk of acquiring HIV to ensure that MHS improvements reflect a broader range of stakeholder priorities. Our multi-disciplinary team is actively addressing this gap through a complementary survey of community members, including people living with HIV, to better understand their views and address the ELSI issues of MHS.

This study demonstrates the utility of choice experiments in systematically exploring improvements to MHS use. The findings reveal that end users, like other stakeholders, desire more evidence on the public health benefits of MHS. Such evidence is essential for assessing whether the trade-offs between benefits and risks are acceptable and for determining whether modifications to MHS practices are warranted. The shared commitment among end users to assess the effectiveness of MHS may be of reassurance to other stakeholders, including scholars, legal experts, and advocates. These findings can inform decision makers seeking to respond to stakeholder concerns and support the ethical use of MHS for public health applications.

## Supplementary information

Supplementary Information

Description of Additional Supplementary Files

Supplementary Data 1 (PDF)

Supplementary Data 2 (Excel)

Reporting Summary

## Data Availability

The source data for Figs. 2, 3, and 4 are available in Supplementary Data 1 (PDF format) and 2 (Excel format), which contain identical content in different file formats for accessibility. The datasets generated by the survey research for the current study are available from the corresponding author upon reasonable request.
