# Supplementary material for: More evidence is needed to improve molecular HIV surveillance for cluster detection and response
Source: Commun Med (Lond). 2025 Nov 14;5:504. doi: 10.1038/s43856-025-01202-0 (PMC12669793; doi:10.1038/s43856-025-01202-0)
Supplement: Supplementary file 1 — Supplementary Information [file 43856_2025_1202_MOESM1_ESM.pdf]

Title: More evidence is needed to improve molecular HIV surveillance for cluster detection and response

Authors: Anne L R Schuster [1§], Juli Bollinger [2], Gail Geller[2,3], Susan J Little[4], Sanjay R Mehta[4], Travis Sanchez[5], Jeremy Sugarman [2,3], John FP Bridges[1,6]

1. Department of Biomedical Informatics, The Ohio State University College of Medicine, Columbus, OH, USA
2. Berman Institute of Bioethics, Johns Hopkins University, Baltimore, MD, USA
3. School of Medicine, Johns Hopkins University, Baltimore, MD, USA
4. Division of Infectious Disease, University of California San Diego, San Diego, CA, USA
5. Department of Epidemiology, Rollins School of Public Health, Emory University, Atlanta, GA, USA
6. Department of Health Behavior and Society, Bloomberg School of Public Health, Johns Hopkins University, Baltimore, MD, USA

[§]**Correspondence to:** anne.schuster@osumc.edu

Supplementary Method

All HIV genetic sequencing data used for MHS in the US originates from blood samples collected from people living with HIV. These samples undergo extraction and amplification of the viral nucleic acids, followed by Sanger sequencing[1] or next-generation sequencing[2] although the CDC does not currently allow public health departments to collect raw next generation sequencing HIV data.[3] Blood samples for MHS are often collected as part of routine clinical care to determine if a person living with HIV has a drug-resistant strain. These results are reported without patient consent to the state public health agency, subsequently stripped of personal identifiers and sent to the national public health surveillance system.[4,5] Researchers may be able to obtain de-identified data from public health departments as part of a data use agreement.[6]

There are two primary approaches to analyzing relationships between HIV genetic sequences for MHS: phylogenetic and clustering approaches.[7] Phylogenetic methods study the evolutionary relationships of organisms based on their genetic similarity using mathematical models. They generate phylogenetic trees (similar to a family tree) to infer an origin—the most recent common ancestors of all sets of sequences in the dataset. Phylogenetic techniques leverage the fact that the viral genetic sequences change over time due to mutations, where some nucleotides are replaced by others due to imprecise copying. These mutations accumulate with time and can be passed on during transmission of the virus to another host. This means that sequences from closely related viruses are more similar than those from more distantly related ones. Correspondingly, viral sequences obtained from individuals who are more closely related along a transmission chain will be more similar to each other. Analyses of these sequences can estimate who transmitted HIV to whom, but they cannot prove direct transmission. Phylogenetic techniques are most often used in research that may help to inform public health initiatives.[8-10]

Clustering approaches aim to group HIV sequences by how similar they are to each other. While the similarity between sequences could be based on their closeness in a phylogenetic tree, clustering approaches typically do not rely on assessing evolutionary patterns. Instead, they measure the nucleotide differences between HIV sequences and characterize closely related sequences as "clusters" of closely linked sequences, arising from a common transmission "network." Clustering approaches essentially

align, compare, and calculate a genetic distance between HIV sequences in a dataset. This process yields a matrix of pairwise differences, from which clusters are identified based on a pre-established genetic distance threshold (e.g., 0.5% for CDC priority clusters). All pairwise differences below or equal to this threshold are considered putative transmission linkages that are not necessarily direct transmission events but identify networks of closely linked (i.e., clustered) transmission events. Results from clustering approaches can be visualized as network diagrams with nodes representing a case and linkages between them represented by lines connecting the nodes. The clustering approach has been primarily used in public health to detect recent and rapid transmission clusters.[4,11-13]

Supplementary Table 1. Attributes and attribute descriptions

Attribute	Attribute description
**Certainty of benefit**	Using HIV molecular epidemiology may help reduce HIV transmission. For example, the use of HIV molecular epidemiology could help identify gaps in prevention services or improve linkage to HIV care. We will consider the level of certainty regarding the evidence on the benefits of HIV molecular epidemiology.
**Data use communication**	Programs that use HIV molecular epidemiology may differ in how they inform individuals about the use of their personal data, including their HIV sequence data. We will consider three communication approaches that vary by how personal they are.
**Type of HIV transmission inference**	Programs using HIV molecular epidemiology can make different types of transmission inferences. The type of inference depends on the data used in the analysis. This includes the kind of HIV molecular sequence data and additional data such as demographic, behavioral, clinical and laboratory data. We will consider three types of transmission inferences based on how personal they could be.
**Depth of HIV sequence sampling**	Proportion of persons living with HIV in a given region with viral sequences available for analysis. The depth of sampling may influence the detectability of networks experiencing rapid HIV transmission. We will consider three levels of sampling depth.
**Risk of stigma**	HIV-related stigma refers to having negative attitudes and beliefs about persons living with HIV or who engage in behaviors that may increase their chances of acquiring HIV. Programs using HIV molecular epidemiology could increase HIV-related stigma depending on how they report or act on results. Increased stigma could discourage people from seeking health care. We will consider three levels of added risk of stigma.

Supplementary Figure 1. Comparison of preferences for researchers and public health practitioners for ways to improve MHS

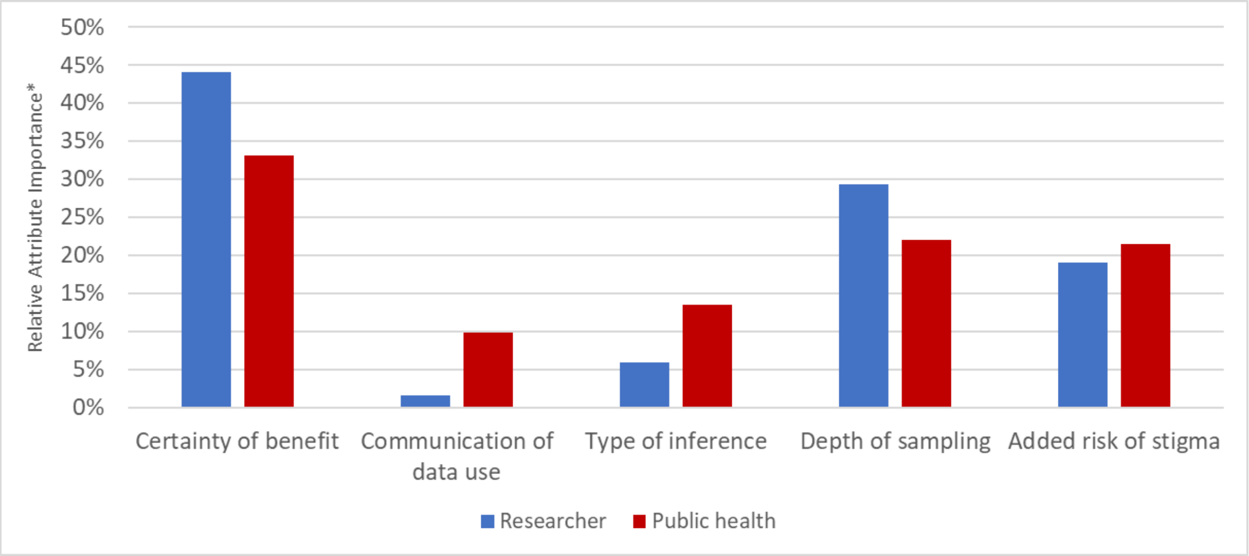

References

1. Sanger F, Nicklen S, Coulson AR. DNA sequencing with chain-terminating inhibitors. *Proc Natl Acad Sci U S A*. Dec 1977;74(12):5463-7. doi:10.1073/pnas.74.12.5463
2. Metzker ML. Sequencing technologies - the next generation. *Nat Rev Genet*. 2010;11(1):31-46. doi:10.1038/nrg2626
3. Centers for Disease Control and Prevention. Expanded guidance on collection, use and release of HIV sequence data. Accessed October 22, 2024. https://www.cdc.gov/hiv/pdf/funding/announcements/ps18-1802/cdc-hiv-sequence-guidance.pdf
4. Oster AM, France AM, Panneer N, et al. Identifying Clusters of Recent and Rapid HIV Transmission Through Analysis of Molecular Surveillance Data. *J Acquir Immune Defic Syndr*. 2018;79(5):543-550. doi:10.1097/qai.0000000000001856
5. Oster AM, Panneer N, Lyss SB, et al. Increasing Capacity to Detect Clusters of Rapid HIV Transmission in Varied Populations—United States. *Viruses*. 2021;13(4):577.
6. Mehta SR, Schairer C, Little S. Ethical issues in HIV phylogenetics and molecular epidemiology. *Curr Opin HIV AIDS*. May 2019;14(3):221-226. doi:10.1097/coh.0000000000000538
7. Pennings PS, Holmes SP, Shafer RW. HIV-1 transmission networks in a small world. *J Infect Dis*. Jan 15 2014;209(2):180-2. doi:10.1093/infdis/jit525
8. Ragonnet-Cronin M, Jackson C, Bradley-Stewart A, et al. Recent and Rapid Transmission of HIV Among People Who Inject Drugs in Scotland Revealed Through Phylogenetic Analysis. *J Infect Dis*. May 25 2018;217(12):1875-1882. doi:10.1093/infdis/jiy130
9. Paraskevis D, Paraschiv S, Sypsa V, et al. Enhanced HIV-1 surveillance using molecular epidemiology to study and monitor HIV-1 outbreaks among intravenous drug users (IDUs) in Athens and Bucharest. *Infect Genet Evol*. 2015;35:109-21. doi:10.1016/j.meegid.2015.08.004
10. Campbell EM, Jia H, Shankar A, et al. Detailed Transmission Network Analysis of a Large Opiate-Driven Outbreak of HIV Infection in the United States. *J Infect Dis*. Nov 27 2017;216(9):1053-1062. doi:10.1093/infdis/jix307
11. Kosakovsky Pond SL, Weaver S, Leigh Brown AJ, Wertheim JO. HIV-TRACE (TRAnsmission Cluster Engine): A tool for large scale molecular epidemiology of HIV-1 and other rapidly evolving pathogens. *Mol Biol Evol*. Jul 1 2018;35(7):1812-1819. doi:10.1093/molbev/msy016
12. Ragonnet-Cronin M, Hu YW, Morris SR, Sheng Z, Poortinga K, Wertheim JO. HIV transmission networks among transgender women in Los Angeles County, CA, USA: a phylogenetic analysis of surveillance data. *Lancet HIV*. 2019;6(3):e164-e172. doi:10.1016/s2352-3018(18)30359-x
13. Wertheim JO, Leigh Brown AJ, Hepler NL, et al. The global transmission network of HIV-1. *J Infect Dis*. 2014;209(2):304-13. doi:10.1093/infdis/jit524
