# Supplementary material for: More evidence is needed to improve molecular HIV surveillance for cluster detection and response
Source: Commun Med (Lond). 2025 Nov 14;5:504. doi: 10.1038/s43856-025-01202-0 (PMC12669793; doi:10.1038/s43856-025-01202-0)
Supplement: Supplementary file 2 — Description of Additional Supplementary Files [file 43856_2025_1202_MOESM2_ESM.pdf]

Description of Additional Supplementary Files

File name: Supplementary Data 1-2

Description: The source data for Figures 2, 3, and 4
