# Supplementary material for: More evidence is needed to improve molecular HIV surveillance for cluster detection and response
Source: Commun Med (Lond). 2025 Nov 14;5:504. doi: 10.1038/s43856-025-01202-0 (PMC12669793; doi:10.1038/s43856-025-01202-0)
Supplement: Supplementary file 3 — Supplementary Data 1 (PDF) [file 43856_2025_1202_MOESM3_ESM.pdf]

Figure 2

	Relative attribute importance
Certainty of benefit	0.39
Data use communication	0.06
Type of transmission inference	0.10
Depth of sampling	0.26
Decrease risk of stigma	0.20

Figure 3

	Relative attribute importance	
	Attitudes	Preferences
Certainty of benefit	0.20	0.39
Data use communication	0.18	0.06
Type of transmission inference	0.17	0.10
Depth of sampling	0.24	0.26
Decrease risk of stigma	0.22	0.20

Figure 4

	Mean		SE	
	Research	Public health	Researsh	Public health
Lack of individual consent	0.444	0.428	-0.1	-0.1
Limits resources for other activities	0.407	0.392	-0.1	-0.09
Re-use data collected for clinical uses	0.37	0.321	-0.09	-0.09
Limited evidence of benefits	0.63	0.57	-0.09	-0.1
Increase risk of individual harm	0.592	0.5	-0.1	-0.1
Lack of opt-out option	0.519	0.392	-0.1	-0.09
Lacks direct data use disclosure	0.63	0.5	-0.09	-0.1
Increase risk of stigma towards groups	0.74	0.535	-0.09	-0.09
Infer HIV transmission directionality	0.703	0.428	-0.09	-0.09
Infer HIV transmission source	0.629	0.321	-0.09	-0.09
Increase the risk of legal prosecution	0.815	0.464	-0.8	-0.1
