# Supplementary material for: More evidence is needed to improve molecular HIV surveillance for cluster detection and response
Source: Commun Med (Lond). 2025 Nov 14;5:504. doi: 10.1038/s43856-025-01202-0 (PMC12669793; doi:10.1038/s43856-025-01202-0)
Supplement: Supplementary file 5 — Reporting Summary [file 43856_2025_1202_MOESM5_ESM.pdf]

nature portfolio

Corresponding author(s): Anne Schuster

Last updated by author(s): Sep 19, 2025

Reporting Summary

Nature Portfolio wishes to improve the reproducibility of the work that we publish. This form provides structure for consistency and transparency in reporting. For further information on Nature Portfolio policies, see our Editorial Policies and the Editorial Policy Checklist.

Statistics

For all statistical analyses, confirm that the following items are present in the figure legend, table legend, main text, or Methods section.

n/a	Confirmed	
☐	☒	The exact sample size (*n*) for each experimental group/condition, given as a discrete number and unit of measurement
☐	☒	A statement on whether measurements were taken from distinct samples or whether the same sample was measured repeatedly
☐	☒	The statistical test(s) used AND whether they are one- or two-sided<br>*Only common tests should be described solely by name; describe more complex techniques in the Methods section.*
☒	☐	A description of all covariates tested
☒	☐	A description of any assumptions or corrections, such as tests of normality and adjustment for multiple comparisons
☐	☒	A full description of the statistical parameters including central tendency (e.g. means) or other basic estimates (e.g. regression coefficient) AND variation (e.g. standard deviation) or associated estimates of uncertainty (e.g. confidence intervals)
☐	☒	For null hypothesis testing, the test statistic (e.g. *F*, *t*, *r*) with confidence intervals, effect sizes, degrees of freedom and *P* value noted<br>*Give P values as exact values whenever suitable.*
☒	☐	For Bayesian analysis, information on the choice of priors and Markov chain Monte Carlo settings
☒	☐	For hierarchical and complex designs, identification of the appropriate level for tests and full reporting of outcomes
☐	☒	Estimates of effect sizes (e.g. Cohen's *d*, Pearson's *r*), indicating how they were calculated

Our web collection on statistics for biologists contains articles on many of the points above.

Software and code

Policy information about availability of computer code

Data collection	Data was collected through Qualtrics (Qualtrics, Provo, UT).
Data analysis	Quantitative analyses were conducted using Stata SE version 17 (StataCorp LLC).

For manuscripts utilizing custom algorithms or software that are central to the research but not yet described in published literature, software must be made available to editors and reviewers. We strongly encourage code deposition in a community repository (e.g. GitHub). See the Nature Portfolio guidelines for submitting code & software for further information.

Data

Policy information about availability of data

All manuscripts must include a data availability statement. This statement should provide the following information, where applicable:

- Accession codes, unique identifiers, or web links for publicly available datasets
- A description of any restrictions on data availability
- For clinical datasets or third party data, please ensure that the statement adheres to our policy

The source data for Figures 2, 3, and 4 are available in Supplementary Data 1. The datasets generated by the survey research for the current study are available from the corresponding author on reasonable request.

Research involving human participants, their data, or biological material

Policy information about studies with human participants or human data. See also policy information about sex, gender (identity/presentation), and sexual orientation and race, ethnicity and racism.

Reporting on sex and gender	n/a
Reporting on race, ethnicity, or other socially relevant groupings	n/a
Population characteristics	Data was collected on end users' primary role (researcher or public health practitioner) and number of years working in their field (<5 years, 5 – 10 years, >10 years).
Recruitment	End users invited to participate in our study included researchers with expertise in MHS and practitioners from U.S. public health departments who had experience with oversight or implementation of MHS for surveillance and/or public health responses to HIV. Participants were identified through their authorship of peer-reviewed publications, presentations at major HIV conferences, leadership roles in national societies/centers, publicly available lists of HIV-focused public health officials, and/or the project's Expert Advisory Board members. All potentially eligible end users were invited to participate in the survey via email. End users who opted-in to participate were sent an individual online survey link administered through Qualtrics (Qualtrics, Provo, UT).
Ethics oversight	The expert engagement was reviewed and deemed non-human subjects research by the Johns Hopkins Medicine Institutional Review Board (reference number IRB00354016) under U.S. Department of Health and Human Services (DHHS) or Food and Drug Administration (FDA) regulations. The conduct of the survey was reviewed and deemed non-human subject research by the Ohio State University College of Medicine Institutional Review Board (reference number 2022E1207) based on these regulations.

Note that full information on the approval of the study protocol must also be provided in the manuscript.

Field-specific reporting

Please select the one below that is the best fit for your research. If you are not sure, read the appropriate sections before making your selection.

☐ Life sciences ☒ Behavioural & social sciences ☐ Ecological, evolutionary & environmental sciences

For a reference copy of the document with all sections, see nature.com/documents/nr-reporting-summary-flat.pdf

Behavioural & social sciences study design

All studies must disclose on these points even when the disclosure is negative.

Study description	The study used a choice experiment to collect quantitative data about end user preferences. It used additional close-ended questions to collect quantitative data about end user attitudes, and used open-ended questions to collect qualitative data to provide additional context.
Research sample	End users included researchers with expertise in MHS and public health practitioners from U.S. public health departments who had experience with oversight or implementation of MHS for surveillance and/or public health responses to HIV.
Sampling strategy	The entire pool of potential end users were invited to participate in the survey, and those who felt capable to respond participated.
Data collection	Data was collected via an online survey administered through Qualtrics.
Timing	The survey was fielded from May to June 2023.
Data exclusions	No data were excluded from the analyses.
Non-participation	We identified 90 potential end-users of whom 55 felt capable to participate and agreed to do so (response rate=61%).
Randomization	Participants were not allotted into experimental groups.

Reporting for specific materials, systems and methods

We require information from authors about some types of materials, experimental systems and methods used in many studies. Here, indicate whether each material, system or method listed is relevant to your study. If you are not sure if a list item applies to your research, read the appropriate section before selecting a response.

Materials & experimental systems

n/a	Involved in the study
☒	☐ Antibodies
☒	☐ Eukaryotic cell lines
☒	☐ Palaeontology and archaeology
☒	☐ Animals and other organisms
☒	☐ Clinical data
☒	☐ Dual use research of concern
☒	☐ Plants

Methods

n/a	Involved in the study
☒	☐ ChIP-seq
☒	☐ Flow cytometry
☒	☐ MRI-based neuroimaging

Plants

Seed stocks: n/a

Novel plant genotypes: n/a

Authentication: n/a
